# Microsaccade Dynamics Enhance Nodule Detection and Discriminate Reader Expertise Levels During Chest Radiography

**DOI:** 10.21203/rs.3.rs-9694768/v1

**Published:** 2026-06-29

**Authors:** Robert G. Alexander, Stephen Waite, Shawn Lyo, Ashwin Venkatakrishnan, Arcadij Grigorian, Riley Morrone, Samuel Sabzanov, Shamsul Alam, Ellis Gayles, Stephen L. Macknik, Susana Martinez-Conde

**Affiliations:** 1 Department of Psychology & Counseling, New York Institute of Technology, USA; 2 Department of Radiology, SUNY Downstate Health Sciences University, USA; 3 Department of Ophthalmology, SUNY Downstate Health Sciences University, USA; 4 Expertize, Inc., USA

**Keywords:** perceptual expertise in radiology, medical image perception, fixational eye movements, microsaccades, saccades, visual search, lung nodule detection, recognition errors, decision-making errors, cancer, perceptual error

## Abstract

**Background:**

Perceptual misses are a major source of diagnostic error in radiology, yet no physiological biomarker currently exists for expertise in chest radiograph interpretation.

**Purpose:**

To determine whether microsaccade dynamics enhance pulmonary nodule detection and discriminate reader expertise levels during chest radiography.

**Materials and Methods:**

In this experimental eye-tracking study, 26 adults (nine naive observers, 11 radiology residents, and six attending radiologists) searched for nodules in 148 chest radiographs from the Japanese Society of Radiological Technology database. Eye movements were recorded during image interpretation. Readers were analyzed by both training level and performance-based accuracy groups. Microsaccade dynamics were compared inside versus outside the nodule region and on correct versus incorrect trials. Receiver operating characteristic analyses were used to test whether oculomotor variables discriminated lower- from higher-accuracy readers.

**Results:**

On correct trials, across expertise levels, readers generated more microsaccades per fixation when viewing nodules than when viewing other image regions. This relationship was absent on incorrect trials, linking microsaccade production to successful abnormality detection. The effect remained after matching fixation durations inside and outside the target region, indicating that it was not explained solely by longer fixation times. Higher-accuracy readers also showed a greater prevalence of fixations containing microsaccades within the target region on correct trials. Receiver operating characteristic analyses identified a microsaccade-based oculomotor signature that differentiated lower- from higher-accuracy readers, including microsaccade rate within the target region on correct trials.

**Conclusion:**

Microsaccade dynamics enhance pulmonary nodule detection during chest radiography and provide, to our knowledge, the first microsaccade-based, largely non-volitional physiological biomarker of expertise in chest radiograph interpretation.

Chest radiograph interpretation is fundamentally a perceptual task. Because abnormality detection is the first step in the diagnostic pathway^1,2^, perceptual misses can prematurely terminate diagnostic workups, resulting in medical error and potential patient harm^3–7^. Radiological search is especially demanding because abnormalities vary widely in appearance^8^, may resemble surrounding anatomy^9–11^, and are often partially obscured by overlapping structures^7,12,13^. Despite these challenges, radiologists develop perceptual skills that permit relatively accurate image interpretation.

Microsaccades contribute to visual processing during fixation and search, but their role in chest radiograph interpretation remains unknown^14–16^. Prior work has shown that microsaccades can support visibility and information acquisition in non-radiological settings, and that they are closely linked to visual attention. Here we asked whether microsaccade dynamics also contribute to pulmonary nodule detection during chest radiography^17^.

A second question motivated the present study. Prior studies in surgery, pathology, anesthesia, and radiology have shown that eye-tracking variables can differ across expertise levels. Those measures are objective in the sense that they are quantitatively recorded with unbiased tools, but they rely on broader gaze behaviors such as fixation allocation, dwell time, scanpath structure, or pupil-linked workload that are relatively susceptible to bias and fundamentally rooted in cognitively driven voluntary strategies. By contrast, microsaccades are fixational eye movements that are largely non-volitional, typically occur outside conscious awareness, and are relatively robust to voluntary modulation during task performance. This makes them a strong candidate as an objective physiological biomarker of expertise.

We therefore set out to test two related hypotheses in chest radiograph interpretation. First, if microsaccades enhance information acquisition during radiological search, they should be more prevalent when readers successfully inspect nodules than when they fail to detect them. Second, if expertise is associated with stable differences in non-volitional oculomotor processing, microsaccade-linked dynamics should systematically differentiate lower- from higher-performing readers. To test these ideas, we recorded the eye movements of naive observers, radiology residents, and attending radiologists while they searched for pulmonary nodules in chest radiographs.

## Methods

### Participants.

26 adults participated in this research and were compensated $15 per session: 9 naive adults with no formal training in radiology diagnostics, 11 radiology residents, and 6 attending radiologists. Because years of training and academic rank do not always map cleanly onto perceptual performance, these participants were re-categorized into “low,” “intermediate,” and “high” accuracy groups based on their radiologic performance accuracy (see the “Defining expertise” Results subsection for details).

All participants self-reported normal or corrected-to-normal vision.

All experiments were performed in accordance with relevant guidelines and regulations. This study was approved by the SUNY Downstate Health Sciences University Institutional Review Board (protocol #1733700). Written informed consent was obtained from all participants.

### Stimuli and apparatus.

Participants rested their forehead and chin on the EyeLink 1000 head/chin support, ~72.0 cm away from a linearized video monitor (Barco Reference Calibrator V, 60 Hz refresh rate).

The images presented in this study were part of the Standard Digital Image Database from the Japanese Society of Radiological Technology (JSRT)^18^. The JSRT dataset includes 154 conventional chest radiographs containing a single lung nodule per image. Here, we displayed 148 of the 154 images (six of the images were omitted due to technical difficulties).

Images in the JSRT dataset are ranked into five groups according to their nodules’ subtlety/obviousness, following consensus by a panel of radiologists (see^18^ for details). Of the 148 images presented in our study, 25 were rated as rank 1 (“extremely subtle”), 28 as rank 2, 47 as rank 3, 36 as rank 4, and 12 as rank 5 (“obvious”).

The JSRT database includes X and Y coordinates for each nodule, which approximate the nodule’s center point. We used these to determine each nodule location and to assign a region of interest, with a 1.5° radius, centered on the X,Y coordinates. We refer hereafter to this region of interest as the “target window.” We visually confirmed that all the nodules in the selected images were contained within the target window.

Images were displayed at a resolution of 1600×1200 pixels. Accordingly, each radiograph was downsampled to 1200×1200 (from their original 2048×2048 size) before being displayed.

### Procedure.

Participants were tasked with searching through each chest radiograph and locating the single nodule present in each image. To indicate the nodule’s position, participants were instructed to press a button on a gamepad while looking directly at the nodule, once located. Each chest radiograph remained on the screen until the participant produced this button-press response. Participants had unlimited time to view each image, but they were asked to respond as quickly as possible without diminishing accuracy. Participants were told that if they could not locate a nodule, they should guess at its most likely location.

Responses were considered correct if, at the time of the button press, the participant’s gaze position was inside the target window. Responses were considered incorrect if, at the time of the button press, the participant’s gaze position was outside the target window. No feedback was provided during the experiment.

Each trial included a single radiograph, and each radiograph appeared just once during the experiment. Radiographs were presented in random order. Participants completed a total of 148 trials (one trial per image) during a single experimental session.

### Eye movement and gaze analyses.

Eye position was acquired noninvasively with a fast video-based eye tracker at 500 Hz (EyeLink 1000, SR Research). We recorded eye movements simultaneously in both eyes, identifying and removing blink periods as portions of the raw data where pupil information was missing. We also removed portions of data where very fast decreases and increases in pupil area occurred (>50 units per sample; such periods might indicate semi-blinks where the pupil is never fully occluded)^19,20^. In addition, we removed 200ms of raw data before and after each blink/semi-blink to eliminate the initial and final parts where the pupil was still partially occluded^19^. Saccades were identified with a modified version of the algorithm developed by Engbert and Kliegl et al.^21–25^, with λ = 6 (used to obtain the velocity threshold) and a minimum saccadic duration of 6 ms. To reduce the amount of potential noise, we considered only binocular saccades, that is, saccades with a minimum overlap of one data sample in both eyes^20,22–25^. Moreover, we imposed a minimum intersaccadic interval of 20 ms so that potential overshoot corrections might not be categorized as new saccades^26^. To calculate microsaccade properties such as magnitude and peak velocity, we averaged the values for the right and left eyes. Microsaccades were defined as saccades with magnitude <1°^15,20,27–29^. **Supplemental Figure 1** shows the peak velocity-magnitude relationship (panels **A,B,C)** and the magnitude distribution (panels **C,D,E**) for microsaccades (<1°) and saccades (≥1°) for all participants.

We determined how long participants directed their gaze inside vs. outside the target window by calculating their dwell time, defined as the cumulative duration of gaze samples falling within a certain image region (in this case, either inside or outside the target window). To estimate how much of a given image was viewed by each participant, we labelled all pixels on the image within 1.5° of each fixation. We then computed the total area (in pixels) that fell within that radius and divided it by the entire 1200×1200 pixel area over which the radiograph was displayed, to create a percentage. We then divided the results by the maximum value of these percentages across all participants, to obtain the normalized “fraction of foveated image area.” We note that this image area includes pixels that are not relevant to radiological analysis, such as those that lie outside of the body. As such, whereas the current work uses the fraction of foveated image area to assess the relative scanning percentages across groups, this measure does not necessarily represent the absolute percentage of the image that was foveated by radiologists.

Because longer fixations provide more time for microsaccade production, we also analyzed microsaccade dynamics for duration-matched fixations, in which we established a 1:1 correspondence between fixations of comparable duration inside and outside the target window following^14^. Single fixations inside the target window were randomly matched to single fixations outside the target window, with both fixation durations being within 10 ms of each other. This resulted in equivalent distributions of fixation durations and numbers of fixations, removing any potential conflation between such factors and microsaccade production. Then, we analyzed again the prevalence of fixations containing microsaccades inside and outside the target window. We conducted this particular analysis for correct trials only, given the relatively low number of fixations inside the target window on incorrect trials.

### ROC analyses.

We used receiver operating characteristic (ROC) analyses to develop an objective classifier for oculomotor expertise based on the oculomotor behaviors of low-accuracy participants vs. those of intermediate- and high-accuracy participants. For the purpose of this analysis, intermediate and high accuracy participants were combined to achieve sufficient N for pairwise ROC comparisons to be computed accurately.

The area under the ROC curve is directly related to the overlap of the two distributions of the property that is being compared^30^. Thus, the area under each ROC curve provides a measure of the discriminability of two signals, and indicates how well each property serves to discriminate between the classes^30–32^. In this approach, an area of 0.5 corresponds to completely overlapping distributions and an area of 1 corresponds to distributions that can be perfectly discriminated based on that property.

We assessed the statistical significance of the observed area under the ROC curve (AUC) using a permutation procedure^30,33–36^. Group labels (Low vs. Intermediate+High) were randomly shuffled n = 5000 times to generate a null distribution of AUC values, and the p-value was calculated as the proportion of permuted AUCs greater than or equal to the observed AUC, with significance set at *p* < 0.05. The 95th percentile of the permutation distribution served as the cutoff AUC for comparison with the empirical value.

Our analysis also provides a threshold (“optimal working point”) on each ROC curve. To establish the optimal working point, we applied the minimum Euclidean distance method^32,37^. Specifically, the point was defined as the threshold that minimized the distance to the ideal classifier (FPR = 0, TPR = 1):

$$\text{d}=\sqrt{{\left(\text{FPR}-0\right)}^{2}+{\left(\text{TPR}-1\right)}^{2}}$$

This method balances sensitivity (TPR) and specificity (1 – FPR) without requiring explicit assumptions about cost ratios or prevalence. In the present context, the resulting threshold provides an interpretable cutoff distinguishing the group of participants with radiology training (Intermediate+High) from that with no training (Low).

### Consistently vs. inconsistently fixated regions.

Following McCamy, et al.^14^, we determined the image regions most frequently foveated across observers as a proxy for informativeness. To ascertain these regions, we first created fixation heatmaps for each trial and participant^14,16^. First, to simulate foveal range, we created individual fixation maps by convolving Gaussian kernels, with σ = 0.64° for a half-width height of 1.5° (77 pixels), with each fixated location for each image and each participant. Then, we normalized these fixation heatmaps, as follows: $F(i,j)=\left[f(i,j)-\underset{i,j}{\text{min}}(f(i,j))\right]/\left[\underset{i,j}{\text{max}}(f(i,j))-\underset{i,j}{\text{min}}(f(i,j))\right].$

Next, we averaged the normalized fixation heat maps of all but one participant per group (i.e., low accuracy participants). For **Supplemental Figure 6**, this was done separately for images with difficulty rankings of 1, 3, or 5. For **Supplemental Figure 7**, averages were computed across all trials irrespective of image difficulty.

The averaged heat maps were then thresholded using Otsu’s method^38^: the heat map is assumed to contain two classes of pixels, corresponding to consistently fixated (Ψ, the region of pixels above threshold) and inconsistently fixated regions (Ω, the region of pixels below threshold). An exhaustive search is applied to calculate the optimum threshold used to separate the two classes so that their combined spread (sum of within-class variances) is minimal.

The resulting heatmaps provide a visualization of regions that participants found most informative based on their gaze behavior, and how their oculomotor strategy changed as a function of expertise and search difficulty.

### Statistical methods.

Except where otherwise noted, we conducted three-way repeated measures ANOVAs and two-tailed paired t-tests. Significance levels were set to α = 0.05 throughout.

## Results

We used receiver operating characteristic analyses to test whether microsaccade-linked oculomotor variables could discriminate reader expertise levels. Our goal was to determine whether the non-volitional fixational eye-movement dynamics were linked to successful nodule detection and, if so, would serve as an advance in our understanding of visual perception during image reading. Further, if so, microsaccades could be utilized as a physiological signature of expertise during chest radiograph interpretation.

### Defining expertise.

Some participants performed better than one might expect from their experience level alone (i.e. naive, resident, or attending)—see **Supplemental Figure 2** for the accuracy and response times of individual participants. For example, some residents detected pulmonary nodules with higher accuracy than some attendings. This is in line with prior observations that years of experience and academic rank are often inaccurate indicators of radiological performance^3,4,39^.

Improved or worsened performance than might be predicted from a participant’s career level may result from individual differences in perceptual skills. Some observers could possess inherently superior ability to perform visual searches in general, or radiological searches in particular, irrespective of their years of training. In addition, attending radiologists often specialize in a particular arena of radiology and are not ‘expert’ in the full gambit of imaging modalities (i.e. a mammographer may not routinely interpret abdominal CT scans). Further, performance might *decline* for some individuals over time as a function of aging and other factors^40^.

**Figure 1:**
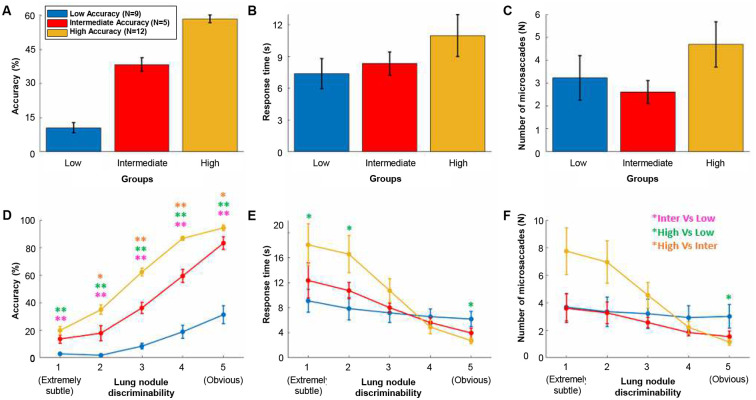
Detection accuracy, response time, and microsaccade production. Mean detection accuracy **(A),** response time **(B)**, and number of microsaccades per trial **(C)** for the low, intermediate, and high accuracy groups. **(D)** High accuracy participants significantly outperformed those in the other two groups across all image difficulty rankings, except for the most subtle images (1: extremely subtle). **(E)** High accuracy participants were faster than other participants when identifying obvious abnormalities, and relatively slow only for the more subtle images. Yet, the overall response time did not significantly differ across the different accuracy groups. **(F)** High accuracy participants produced fewer microsaccades per trial than those in the other groups when searching for obvious lung nodules. * indicates p < .05; ** indicates p < .01. Significance is not indicated in (**A**) because performance accuracy was inherent to how the groups were defined.

To circumvent these issues in the present study, we defined expertise as a function of performance level (i.e. accuracy in radiological search) rather than as a function of an individual’s academic/training rank. Therefore, we did not rely on *a priory* assumptions that attending radiologists (or the equivalent) are more expert in terms of their perceptual accuracy than residents. Rather, for the purpose of the current research, we operationalized expertise as the relative level of nodule detection accuracy among participants, identifying high accuracy participants as “more expert” than low accuracy participants. Accordingly, we used Jenks natural breaks algorithm^41^ to assign each individual to one of three accuracy groups (“low,” “intermediate,” or “high”) to optimize the within-group distances. As a result, participants were re-grouped into “low accuracy” (<25% accurate on average; N=9), “intermediate accuracy” (<50%; N=5), and “high accuracy” groups (the remaining participants; N=12)—see Figure 1A and **Supplemental Figure 3**. We note that such accuracy levels are specific to the search task conducted, and therefore intrinsically arbitrary. Nevertheless, the groupings are highly informative for between-group measures and so all ensuing analyses were conducted using these three accuracy groups. Throughout the manuscript, we use the term “expert” interchangeably with “high accuracy participant.” This clarification notwithstanding, we obtained comparable results when we grouped participants by academic rank—see **Supplemental Figure 4**.

As one might expect, high accuracy participants outperformed those in the other groups not merely in a general sense, but at every image difficulty ranking—see Figure 1D. Conversely, low accuracy participants were surpassed by those in the other two groups at every image difficulty ranking.

### Response times.

Overall response times did not significantly differ across the three accuracy groups, *F*(25)=1.18, *p*=.33—see Figure 1B. However, our analyses revealed an interaction between response times and lung nodule discriminability: high accuracy participants were relatively faster than low accuracy participants on trials where abnormalities were obvious (*p*<.01), and relatively slower on trials where abnormalities were extremely subtle (*p*<.05)—see Figure 1E.

### Foveated Image area.

**Figure 2:**
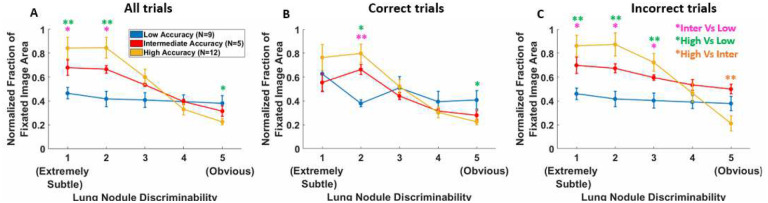
Fraction of image area foveated. **(A)** High accuracy participants viewed a smaller portion of the image on easy trials, and a larger portion of the image on difficult trials, relative to participants in the other groups. This pattern held true both for correct trials **(B)** and incorrect trials **(C)**. * indicates p < .05; ** indicates p < .01. See **Supplemental Figure 5** for the same data before normalization.

On average, high accuracy participants foveated a greater portion of the image than participants in the other groups, *F*(2,115)= 4.8, *p*<.01. Further, image difficulty affected how much of the image was viewed: overall, participants viewed a smaller portion of the image on easy trials, and a greater portion on difficult trials, *F*(4,115)= 2.99, *p*<.05—see Figure 2 and **Supplemental Figure 5**.

For a visualization and further analysis of the viewing patterns for each accuracy group, see **Supplemental Figures 6 and 7**.

### Fixation dynamics.

We assessed gaze dynamics for the three groups of participants, focusing on the eye movements produced while looking directly at the target abnormalities (i.e. “inside” the target window) vs. other parts of the image (i.e. “outside” the target window)—see **Supplemental Tables 1** and **2**.

**Figure 3:**
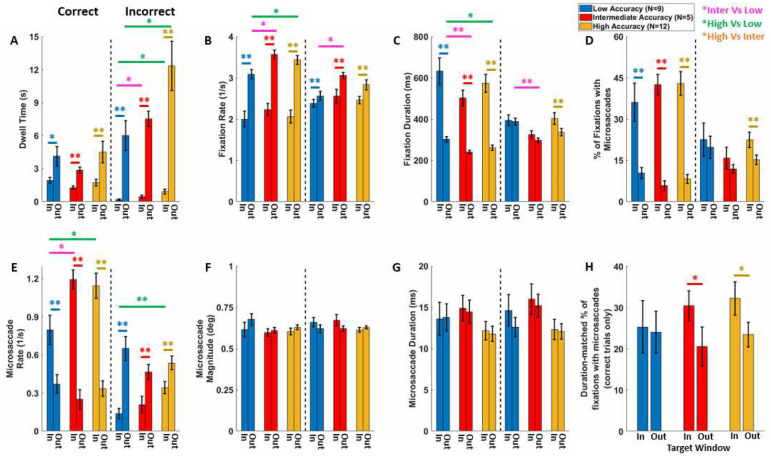
Gaze dynamics inside the target window vs. elsewhere in the image. **(A)** Participants in all groups spent more time viewing the regions outside than inside the target window. High accuracy participants spent the longest time outside of the window on incorrect trials. **(B)** Fixation rates were lower inside than outside the target window, consistent with their correspondingly lower dwell times. **(C)** Fixation durations were longer inside than outside the target window in all groups, especially on correct trials. **(D)** Fixations with microsaccades were more prevalent inside than outside the target window on correct trials in all groups. **(E)** Microsaccade rate was greater inside than outside the target window in correct trials in all groups. Further, on correct trials, low accuracy participants had lower microsaccade rates inside the target window than participants in the other two groups. There were no reliable differences in microsaccade magnitude **(F)** or duration **(G)** as a function of accuracy level, correct/incorrect target identification, or gaze location inside/outside the target window. After matching fixations of equivalent durations, fixations inside the target window remained more likely to contain microsaccades than fixations outside the target window **(H)**, showing that increased fixation durations were not the only driver of increased microsaccade production inside the target window. * indicates p < .05; ** indicates p < .01. See **Supplemental Figure 4** for the same data grouped by rank/career level rather than by accuracy level.

Participants spent less time gazing inside than outside the target window *F*(1,92) = 54.93, *p* < 0.0001—see Figure 3A. This was expected, given that the target window was much smaller than the rest of the image. In other words, participants spent most of their time in a trial searching for abnormalities, as opposed to looking directly at them. However, dwell times differed significantly across the accuracy groups both inside and outside the target window, *F*(2,92) = 3.89, *p* < 0.05. These differences were driven by incorrect trials, on which low accuracy participants spent less time overall viewing the image than both intermediate and high accuracy participants, *p*s < 0.05. This finding was consistent with the low accuracy participants’ relatively fast response times.

Fixation rates were lower inside than outside the target window, consistent with the correspondingly lower dwell times inside than outside the window, *F*(1,92) = 95.91, *p* < 0.0001—see Figure 3B. Taken together, this pattern suggests that high accuracy participants were more persistent on their searches (and thus less likely to guess after a brief scan) than participants in the other groups. Consistent with this interpretation, we also found that low accuracy participants, who struggled to identify even “obvious” abnormalities (Figure 1D), maintained relatively constant response times across image categories (Figure 1E), suggesting that they tended to guess after about eight seconds, irrespective of the difficulty of the image displayed.

Fixation durations were *longer* inside than outside the target window for all groups (see Figure 3C)*, F*(1,92) = 68.99, *p*<.0001, indicating an intense scrutiny of the region around the target once participants’ gaze fell in that area. Likewise, fixation durations were longer within informative (i.e. consistently fixated) than within noninformative (i.e. inconsistently fixated) image regions (see **Supplemental Figure 7B**).

### Microsaccade production.

The total number of microsaccades produced was comparable across accuracy groups, *F*(2,23) = 1.11, *p* = 0.34—see Figure 1C and **Supplemental Table 1**—as was the average microsaccade rate, *F*(2,92) = 1.99, *p* = 0.14. Though microsaccade rates were higher overall on correct than on incorrect trials, *F*(1,92) = 36.33, *p <* 0.0001, we found no differences as a function of the interaction between participant grouping and correct/incorrect reports, *F*(2,92) = 1.25, *p* = 0.29—see Figure 3E.

On correct trials, microsaccade rates were higher inside than outside the target window for each accuracy group, *p*s < 0.0001, pointing to an elevated microsaccade production during the examination of informative, task-relevant portions of the image. Similarly, on correct trials, the percentage of fixations containing microsaccades was higher inside than outside the target window, *F*(1,38) = 43.1, *p <* 0.0001—see Figure 3D. We also observed a significant interaction between participant grouping and correct/incorrect reports regarding the percentage of fixations containing microsaccades, *F*(2,76) = 18.49, *p* < 0.00001. Namely, the percentage of fixations containing microsaccades generally increased with detection accuracy on correct trials, whereas it generally decreased with detection accuracy on incorrect trials.

Microsaccade rates were moreover higher in consistently than in inconsistently fixated regions (see **Supplemental Figure 7D-H**), presumably due to the frequent overlap between the consistently fixated areas and the target window.

Though microsaccade rate analyses (Figure 3E) suggested that this would not be the case, we considered whether increased microsaccade production inside the target window (Figure 3D) could be fully explained by longer fixation durations in the same image regions (Figure 3C). Thus, to de-conflate microsaccade production from fixation duration, we compared microsaccade production across duration-matched gaze fixations inside and outside the target window see also^14^. We found that fixations inside the target window were more likely to contain microsaccades than fixations outside of the target window, even when fixation durations and number of fixations were held equal in both image regions (see Figure 3H). Our combined findings therefore rule out the possibility that increased microsaccade production inside the target window was solely due to more numerous and/or longer gaze fixations than those outside the window. Thus, across all accuracy groups, microsaccades were more prevalent when the observer’s gaze fixation was near the targets. Other microsaccade parameters (such as magnitude and duration) did not differ across accuracy groups (Figure 3F and 3G). These overall results held steady when participants were grouped by career stage rather than by accuracy level—see **Supplemental Figure 4**.

### Towards an oculomotor profile of radiological expertise.

We used receiver operating characteristic (ROC) analyses to determine a profile of consistent oculomotor behaviors that might be used to discriminate across accuracy groups. To do this, we examined the contribution of multiple oculomotor kinematics towards the True Positive Rate (TPR) as a function of the False Positive Rate (FPR) in the nodule detection task, to ascertain a potential oculomotor signature of radiological expertise.

**Figure 4:**
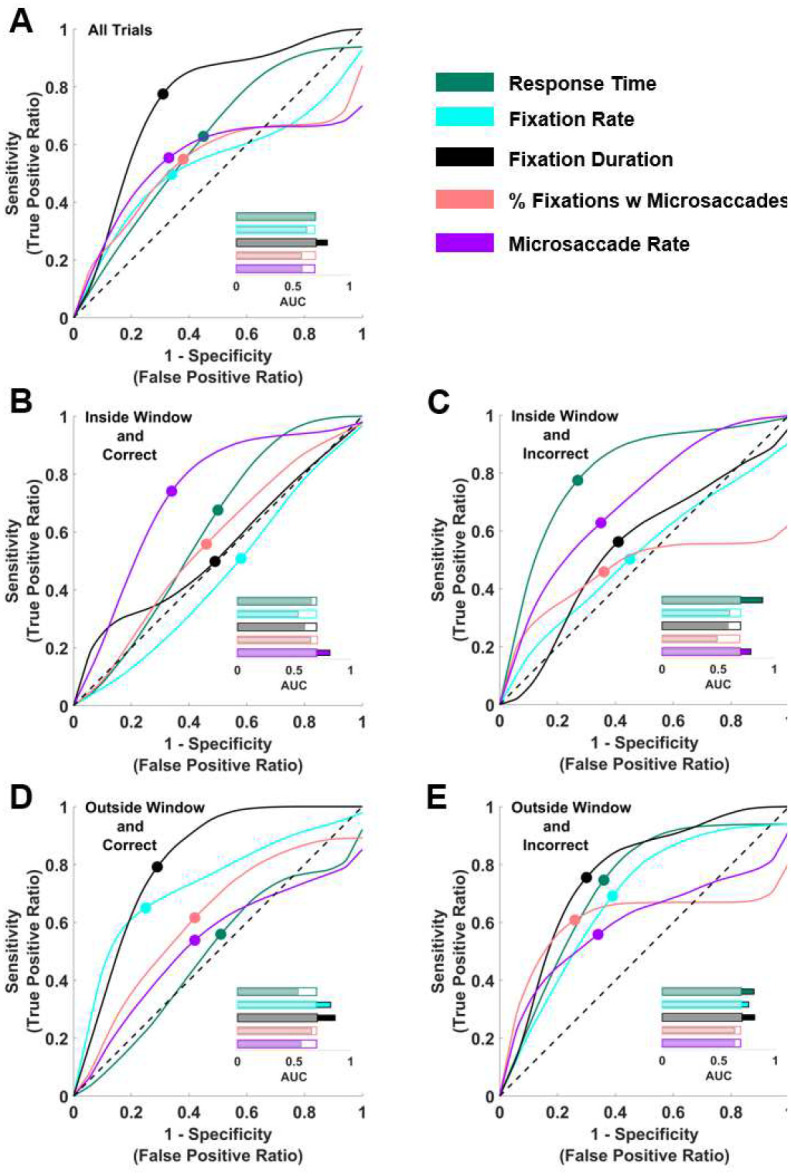
ROC analysis. We used ROC analyses to evaluate the performance of multiple oculomotor properties as a test to discriminate the low accuracy participants (N=9) from the intermediate and high accuracy participants (N=17). Each curve represents the ratio of True Positive Rate/False Positive Rate (discriminability success rate) for a given property. The area under each ROC curve serves a measurement of the discriminator’s performance (where 1 indicates perfect discrimination and 0.5 indicates chance level discrimination). The optimal working point in each curve (point of minimal distance to perfect discriminator performance) is represented with a solid circle. Inset: Area Under the ROC Curve (AUC) for each comparison. The solid bars represent the area under the curve; the empty bars indicate the level necessary to reach *p*≤0.05 significance over chance (determined by permutation analysis). **A)** ROC curves for all trials, irrespective of whether the fixations were inside or outside of the window. Actual AUC values were greater than or equal to the cutoff AUC for response time (AUC_actual_=0.70, AUC_cutoff_ =0.70, *p*<.05, CI_95%_=[0.45,0.92]) and fixation duration (AUC_actual_=0.80, AUC_cutoff_ =0.71, *p*<.005, CI_95%_=[0.59,0.96]). **B)** ROC curves for all fixations inside of the window for correct trials. The analysis was significant for microsaccade rate (AUC_actual_=0.82, AUC_cutoff_ =0.70, *p*<.005, CI_95%_=[0.56,0.99]). **C)** ROC curves for all fixations inside of the window for incorrect trials. The analysis was significant for response time (AUC_actual_=0.90, AUC_cutoff_ =0.69, *p*<.001, CI_95%_=[0.74,1]) and for microsaccade rate (AUC_actual_=0.79, AUC_cutoff_ =.70, *p*<.01, CI_95%_=[0.58,0.95]). **D)** ROC curves for all fixations outside of the window for correct trials. Values were significant for fixation rate (AUC_actual_=0.82, AUC_cutoff_ =0.70, *p*<.01, CI_95%_=[0.64,0.95]) and fixation duration (AUC_actual_=0.86, AUC_cutoff_ =0.71, *p*=.001, CI_95%_=[0.69,0.98]). **E)** ROC curves for all fixations outside of the window for incorrect trials. Values were significant for response time (AUC_actual_=0.81, AUC_cutoff_ =0.70, *p*<.005, CI_95%_=[0.58,1]), fixation rate (AUC_actual_=0.76, AUC_cutoff_ =0.70, *p*<.05, CI_95%_=[0.53,0.96]) and fixation duration (AUC_actual_=0.82, AUC_cutoff_ =0.70, *p*<.005, CI_95%_=[0.62,0.96]).

Specifically, we applied ROC analyses to determine the success rate of a subset of features (i.e. dwell time, fixation rate, fixation duration, percent of fixations with microsaccade, microsaccade rate, microsaccade magnitude, and microsaccade duration) in discriminating low-accuracy participants from intermediate and high accuracy participants (Figure 4), using the area under the ROC curve as a measure of the performance of these properties as discriminators^30–32^.

To obtain the ROC curves, we plotted the probability of true positives vs. the probability of false positives for pairs of subjects across the low accuracy vs. the intermediate- and high-accuracy groups. Our results are consistent with the existence of an oculomotor pattern, which—despite being agnostic to an observer’s success in radiologic searches—can serve as an objective classifier of radiological expertise.

## Discussion

Despite recent advances in understanding how experts and novices allocate their gaze while viewing medical images (e.g.,^42–48^, major gaps remain in our knowledge of how radiologists develop perceptual and oculomotor expertise^3,49^. One notable difference between expert and novice radiologists is that experts make fewer eye movements before foveating an abnormality^50,51^, potentially due to their superior peripheral visual processing of medical images. Yet, the role of gaze behavior on *foveal* visual processing during medical image searches—and its impact of radiological expertise—remains largely unknown.

A key distinction of the present findings is the nature of the oculomotor signal used to index expertise. Prior work in surgery, pathology, and radiology has shown that broader eye-tracking variables can differentiate novice and expert performance. Those measures are objective in the sense that they are quantitatively recorded, but they generally rely on gaze behaviors such as fixation allocation, dwell time, scanpath structure, or pupil-linked workload that are more susceptible to bias and voluntary strategy. By contrast, microsaccades are fixational eye movements that are largely non-volitional, typically occur outside awareness, and are relatively robust to voluntary modulation during task performance. The expertise signature identified here therefore differs from prior gaze-based measures by being rooted in a fixational physiological signal that is less strategically alterable by the observer^52^.

### Microsaccades in radiological search.

Microsaccade production has been linked to the acquisition and processing of relevant visual information in non-radiological tasks^14–16,53^. For instance, McCamy et al. found microsaccades to be more prevalent within informative regions of natural scenes^14^. Otero-Millan et al. also found higher rates of microsaccades near search targets during *Where’s Waldo* visual searches^15^. Here we set out to determine if microsaccade patterns during foveal fixation are indicative of detection accuracy and/or expertise in radiology. Specifically, we asked whether microsaccade kinematics are involved in detecting and reporting lung abnormalities in chest x-rays, and whether microsaccade production changes as a function of expertise on that task. By comparing the microsaccade dynamics of experts and novices near nodules vs. other image areas, we thus aimed to identify specific oculomotor and visual processing strategies linked to diagnostic accuracy.

One outcome of our study might have been that experts produce more microsaccades, supporting their deeper processing of image regions. Alternatively, experts might produce fewer microsaccades, reflecting more efficient task completion with less need for intense foveal scrutiny. Or microsaccade production might not change with expertise, suggesting it is not a modifiable factor in medical image search.

A related question is whether microsaccades can enhance nodule detection. If so, scanpaths with more microsaccades near abnormalities should yield more correct detections than scanpaths without microsaccades near abnormalities.

Our results revealed that experts do not make more microsaccades *overall*, but increased microsaccade production was nevertheless linked to successful nodule detection. These combined findings suggest that microsaccades enhance one’s ability to detect radiological abnormalities, and moreover that radiology experts deploy microsaccades efficiently and selectively while foveating abnormal image regions.

Though participants produced more microsaccades per fixation when foveating abnormalities (Figure 3D,E), these increased microsaccade rates were not fully explained by increased fixation durations (Figure 3H), consistent with the proposal that microsaccades help acquire task-relevant information from informative image regions^14,15^.

Taken together with prior evidence that microsaccades enhance visual processing^15,28,54,55^, our current results suggest that microsaccades may improve target detection across a variety of search tasks, as opposed to specifically enhancing radiological performance during medical image searches.

One potential caveat of our experimental design is that participants reported a nodule’s position by pressing a button while looking directly at the nodule. Thus, elevated microsaccade production within the target window might be potentially related to the participants’ attempt to precisely allocate their gaze for their button-press response—rather than to the target detection itself.

This seemed unlikely given prior findings from non-radiological searches, in which microsaccade rates increased near targets even participants were not asked to foveate the target to make a response^15^. Nevertheless, to rule out this possibility, we repeated the same analyses described above, this time excluding the final fixation from each trial (and therefore removing the microsaccades that directly preceded target detection). There was no substantial change to our results, indicating that this potential confound did not drive our findings—see **Supplemental Figure 8**.

### Microsaccades and radiological expertise.

A previous study found an overall reduction in microsaccade numbers when naive observers received training to detect cancer in mammograms (Hegdé^17^. In contrast, we did not find a significant difference in microsaccade count across expertise levels. In fact, the microsaccade count of high-accuracy participants was numerically (though not significantly) higher than that in the other groups—see Figure 1C and **Supplemental Table 1**. This discrepancy might be due to a confound between trial duration and microsaccade count. That is, because experts generally complete radiologic searches faster than nonexperts, their available time to produce microsaccades is therefore shorter, resulting in lower microsaccade counts (though not necessarily lower microsaccade rates). Indeed, Hegdé (2020) reported shorter trial durations post-training. In our study, experts produced fewer microsaccades (Figure 1F) and had shorter response times (Figure 1E) than novices when searching for “obvious” nodules. However, experts produced more microsaccades and had longer response times when searching for subtle abnormalities. Yet, when we examined the rates— rather than the total numbers—of microsaccades, we found no overall variation with expertise.

Expertise did modulate other aspects of microsaccade production in our study, such as the percentage of fixations containing microsaccades. Yet, across all participants, relatively few fixations contained microsaccades unless a nodule was being located and scrutinized. Thus, although high accuracy observers did not have higher overall microsaccade rates, our data indicates that they nevertheless relied on oculomotor strategies that enhanced the likelihood of microsaccade generation near nodules.

Previous work has shown that radiologists tend to become faster and more accurate as they gain experience^48,50,51,56,57^; see Alexander et al., 2020 for a review. However, accuracy and speed do not always increase together^58^. In the present study, high-accuracy participants spent more time searching for nodules than low-accuracy participants did. However, this pattern changed with image difficulty: experts spent less time searching for obvious abnormalities, and more time searching for subtle abnormalities, as compared to novices. This interaction showcases the difficulty entailed in determining the optimum viewing time for radiological searches in professional settings. That is, whereas high-accuracy search performance can be achieved relatively quickly in some scenarios, other contexts demand a slower approach and greater persistence see^58 for a broader discussion^.

During *incorrect* trials, high-accuracy participants searched for longer times (Figure 3A) and foveated a larger portion of the image (Figure 2C) than low-accuracy participants—despite being ultimately unable to locate the nodule. This specific finding may have been driven by the participants’ a priori knowledge that a nodule was present in each image. Thus, high-accuracy participants may have persisted in their searches due to being confident in their ability to detect a subtle nodule they knew was present, whereas low-accuracy participants may have terminated their searches earlier due to lower confidence in their own skills. Consistent with this explanation, we found that on correct trials with obvious abnormalities, high-accuracy participants could detect the nodules peripherally and thus searched a smaller portion of the image than low-accuracy participants did (Figure 2B).

We also asked whether microsaccade-linked dynamics can serve as a physiological biomarker of reader expertise during chest radiograph interpretation. Our ROC analyses indicate that they can (Figure 4). This is notable because radiologists are not explicitly trained to deploy particular fixational eye-movement patterns during image search. Instead, the repeated perceptual demands and performance feedback of radiology training appear to shape stable differences in oculomotor behavior that are expressed in a largely non-volitional fixational signal. In that sense, the present findings move beyond showing that experts and novices look differently at images: they identify a microsaccade-based physiological discriminator of reader expertise.

Microsaccades are especially attractive as biomarkers because, under ordinary viewing conditions, they are largely non-volitional fixational eye movements and typically occur outside awareness. Unlike broader gaze behaviors such as dwell time or overt scanpath structure, they are not readily deployed as an intentional performance tactic during image interpretation. For this reason, microsaccade-related signatures provide an unusually informative physiological readout of expertise in chest radiograph interpretation..

A potential limitation of our ROC analysis is our relatively modest sample size (Low: N = 9, Inter+High: N = 17). Even so, our total sample size compares favorably with published guidelines. Hajian-Tilaki (2014) provide sample size tables for ROC analysis in diagnostic test studies that indicating that as few as 18 total participants may be sufficient to estimate sensitivity and specificity with acceptable precision, depending on prevalence and marginal error. While those calculations were derived for classical disease vs. non-disease settings rather than multi-parameter/dimension group comparisons, they nonetheless provide a benchmark suggesting that our study is not too small for exploratory ROC analyses. To further guard against instability due to sample size, we complemented AUC estimates with permutation-based significance testing and confidence intervals. Thus, our approach is consistent with prior methodological recommendations.

### Microsaccades and abnormality detection.

In radiology, false negative errors (i.e. missed detections) after foveating an abnormality are traditionally labelled as either “recognition” errors, where abnormalities are viewed “too briefly” to be properly recognized, or “decision-making” errors, where abnormalities are viewed for an adequate length of time but are still dismissed or unrecognized^59–61^. Our combined findings point to the importance of gaze kinematics beyond viewing time in abnormality detection.

Successful recognition of abnormalities likely requires the effective integration of visual and oculomotor processes, *including* appropriate time to foveate the abnormality. Yet, our results indicate that fixation time, per se, is not sufficient: microsaccade production while foveating the abnormality can be critical to successful detection—especially in the case of subtle findings. It follows that accurate medical image interpretation might depend on generating microsaccades within a critical spatial *and* temporal window, and that interventions that optimize this targeted microsaccade production could help improve radiological performance.

The logic of this proposal ensues from our results: radiologists fundamentally perform an oculomotor search task, but because perception is suppressed during saccades^62,63^, information must be acquired during inter-saccadic fixation periods. Microsaccades help sample the visual image in a fashion equivalent to how sniffing aids olfaction^28^, and are the most significant oculomotor contributor to visual enhancement^55^ and neural activity during fixation^64–66^. The present study investigates and quantifies the key role that microsaccades play in radiological detection, as well as the oculomotor strategies that high-performing readers deploy to maximize the benefits of microsaccades during visual search.

In summary, the present study identifies microsaccades as both a functional component of successful pulmonary nodule detection and a physiological discriminator of reader expertise during chest radiograph interpretation. To our knowledge, this is the first microsaccade-based, largely non-volitional physiological biomarker of expertise in this domain. These findings open a new avenue for studying, tracking, and potentially assessing radiological expertise through a physiological signal that is less susceptible to voluntary strategy than broader gaze-based measures.

## Supplementary Files

This is a list of supplementary files associated with this preprint. Click to download.

- RadiologistMicrosaccademay122026SupplementaryInfo.docx

## Data Availability

Upon acceptance of this manuscript, the datasets generated and analyzed during the current study will be deposited in a publicly accessible repository (e.g., crcns.org) and assigned a DOI. All raw and processed data, together with analysis scripts and supporting materials, will be made available at that DOI. Prior to repository release, materials can be obtained from the corresponding author upon reasonable request.
